# Evaluation of Antibody–Drug Conjugate Performances Using a Novel HPLC–DAD Method for Tumor-Specific Detection of DM4 and *S*‑Methyl-DM4

**DOI:** 10.1021/acsomega.5c07693

**Published:** 2025-10-15

**Authors:** Giulio Lovato, Miryam Perrucci, Ilaria Cela, Alessia Lamolinara, Arianna Mercatelli, Vincenzo De Laurenzi, Emily Capone, Marcello Locatelli, Gianluca Sala

**Affiliations:** † Department of Innovative Technologies in Medicine and Dentistry, Center for Advanced Studies and Technology (CAST), University of Chieti-Pescara “G. D’Annunzio”, Via dei Vestini 31, Chieti 66100, Italy; ‡ Center for Advanced Studies and Technology (CAST), University of Chieti-Pescara “G. D’Annunzio”, Via dei Vestini 31, Chieti 66100, Italy; § Department of Biosciences and Agro-Food and Environmental Technologies, 19031 University of Teramo, Teramo 64100, Italy; ∥ Department of Medicine and Aging Sciences, G. D’Annunzio University of Chieti-Pescara, Chieti 66100, Italy; ⊥ Department of Science, 9301 University of Chieti-Pescara “G. D’Annunzio”, Chieti 66100, Italy

## Abstract

Antibody–drug conjugates (ADCs) are an emerging class of therapeutics that have gained interest in precision medicine for cancer treatment, combining the targeted delivery capabilities of monoclonal antibodies with the potent cytotoxicity of small-molecule drugs. The goal is to enhance the therapeutic window by maximizing tumor cell killing while minimizing off-target toxicity. Pivotal aims are precise delivery of payload in the tumor site and a robust analytical methodology to quantify the accumulation of it. 1959-sss/DM4, a novel ADC targeting highly glycosylated LGALS3BP, which is a protein implicated in tumor progression and metastasis, was developed. This ADC has shown potent and durable antitumor activity in various preclinical models. A high-performance liquid chromatography (HPLC) method according to ICH guidelines for the simultaneous quantification of DM4 and its primary metabolite, *S*-methyl-DM4, in tissue matrices was validated. Was employed a patient-derived neuroblastoma cell line (hNB CTRL), which naturally lacks LGALS3BP expression, alongside a counterpart cell line (hNB LGALS3BP) in which LGALS3BP expression was ectopically induced via lentiviral-mediated gene transduction. A second metastatic model using SKNAS neuroblastoma cells, which endogenously express LGALS3BP, was utilized to study the kinetics of the payload. The approach allows for the simultaneous detection of DM4 and *S*-methyl-DM4, offering a valuable tool for the comparative assessment of ADC performance in preclinical models.

## Graphical abstract

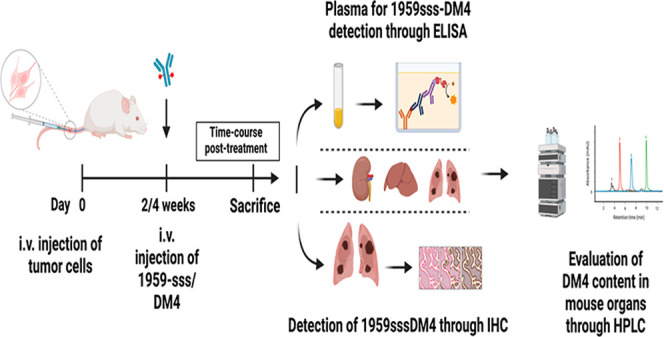

## 1 Introduction

Antibody–drug conjugates (ADCs) have emerged as an innovative oncology therapy by combining the selectivity of monoclonal antibodies (mAb) with the potent cytotoxicity of small-molecule drugs.^1–4^ This strategy enables the targeted delivery of highly toxic agents directly to tumor cells, thereby minimizing damage to normal tissues, a concept validated by several approved ADCs and numerous candidates currently under clinical evaluation.^5–7^ An attractive target for ADC therapy is LGALS3BP, a heavy glycosylated protein that resulted to be overexpressed in a wide range of malignancies and implicated in tumor progression, metastasis, and immune modulation.^8^ Its abundant secretion into the tumor microenvironment supports the use of a noninternalizing ADC strategy, where the therapeutic antibody accumulates in the tumor milieu and locally releases its cytotoxic payload.^4,9^

This agent utilizes a humanized therapeutic anti-LGALS3BP antibody (1959) that has been engineered by substituting key cysteine with serine residues to allow precise, site-specific conjugation of the maytansinoid DM4 via a disulfide linker.^9–11^ This strategy achieves a well-defined drug-to-antibody ratio while preserving high affinity for LGALS3BP. Upon systemic administration, 1959-sss/DM4 selectively localizes to LGALS3BP-positive tumors. In the reducing tumor environment, the disulfide bond is cleaved to release DM4. Owing to its hydrophobicity, DM4 readily crosses cell membranes, where it binds to tubulin, disrupts microtubule dynamics, induces cell cycle arrest, and triggers apoptosis.

This ADC is endowed with a potent and durable antitumor activity, as evaluated in several preclinical models using different cancer models.^12–15^ Furthermore, this ADC, when labeled with Zirconium-89, effectively visualized LGALS3BP-secreting tumors in mouse models using positron emission tomography (PET) imaging.^16^

Notably, DM4 undergoes metabolic transformation (*S*-methylation) to yield *S*-methyl-DM4, a metabolite that demonstrates notable cytotoxicity as its parental drug.^17,18^ In addition to its direct cytotoxic effect, the ADC’s efficacy is further amplified by the “bystander effect”.^19,20^ This phenomenon allows the released DM4 and *S*-methyl-DM4 to diffuse into adjacent tumor cells and cancer-associated cells (such as cancer-associated fibroblasts, immune cells, and adipocytes) that may lack or express low levels of LGALS3BP, thereby broadening the antitumor response. However, this beneficial bystander killing may also lead to off-target toxicity if free DM4 or its metabolites accumulate in normal tissues. Hence, comprehensive pharmacokinetic and biodistribution studies of both DM4 and *S*-methyl-DM4 are essential for optimizing the therapeutic window of DM4-based ADCs. Traditional mass spectrometry-based methods offer high sensitivity and accuracy; however, their high cost and requirement for specialized expertise often restrict widespread adoption.^20,21^ To address these limitations, our study presents a novel high-performance liquid chromatography (HPLC) method coupled with diode array detection (DAD) that enables the simultaneous quantification of DM4 and its primary metabolite, *S*-methyl-DM4, in biological matrices. We applied this method to tissue samples from pseudometastatic neuroblastoma models, previously validated in preclinical studies, to comprehensively characterize the pharmacokinetics and biodistribution of DM4 released from the ADC. This approach further enables us to evaluate the stability and tumor-targeting efficiency of 1959-sss/DM4 while elucidating the relationship between payload release, metabolic conversion, and antitumor efficacy.

## 2 Materials and Methods

### 2.1 Cell Lines and Biochemicals

Human neuroblastoma SKNAS cells were purchased from the American Type Culture Collection (ATCC; Rockville, MD, USA). The primary human neuroblastoma hNB cell line was isolated from a non-MYCN amplified tumor metastasized in the neck of a 3 year old male patient. The 11q and ALK status is not known.^12^ SKNAS were grown in Dulbecco’s modified Eagle’s medium (DMEM, Gibco; Whitman, MA, USA), while stable transduced hNB CTRL and LGALS3BP were grown in Roswell Park Memorial Institute (RPMI) 1640 (Gibco). Human ovarian epithelial OVCAR-3 cells were obtained from the American Type Culture Collection (ATCC; Rockville, MD, USA) and cultured in Dulbecco’s modified Eagle’s medium (DMEM: Gibco, Waltham, MA, USA). All media were supplemented with 10% heat-inactivated fetal bovine serum (FBS) (Gibco), 100 U/mL penicillin, and 100 μg/mL streptomycin (Sigma-Aldrich Corporation, St. Louis, MO, USA). All cells were maintained at 37 °C in a humified air with 5% CO_2_. All cell lines were tested for mycoplasma contamination through the polymerase chain reaction (PCR).

### 2.2 Chemicals

For high-performance liquid chromatography (HPLC) analysis, Ravtansine (DM4) and its primary metabolite, *S*-methyl-DM4 (*S*-Met-DM4), were sourced from MedChemExpress (New Jersey, USA). *N*,*N*-dimethylacetamide (DMA) was obtained from Sigma-Aldrich (St. Louis, MO, USA). Acetonitrile (AcN) and formic acid, both HPLC grade, were supplied by Carlo Erba Reagents (Milan, Italy). Milli-Q purified water (Merck, Darmstadt, Germany) was used for all procedures requiring high-purity water. Purified 1959-sss was purchased from EVITRIA (Schlieren, Switzerland).

The lysis buffer was prepared with 6 M urea, 100 mM Tris-base, 2% CHAPS, 1% Triton X-100, and 50 mM DTT (pH 7.5), with all reagents obtained from Merck. The extraction solvent consisted of ethyl acetate and methanol in a 7.5:1 (*v:v*) ratio, both purchased from VWR Chemicals (Pennsylvania, USA). *N*-Ethylmaleimide (NEM) and ethyl butyl ether were also obtained from Merck. Phosphate-buffered saline (PBS; Oxoid) was prepared by dissolving one tablet per 100 mL of purified water. Ammonium sulfate and ammonium acetate were acquired from PanReac (Barcellona, Spain). Mirvetuximab soravtansine (IMGN853) solution was also obtained from MedChemExpress (New Jersey, USA).

### 2.3 1959-sss/DM4 Purification and Characterization

1959-sss/DM4 is an ADC currently in preclinical development by Mediapharma Srl. Here, it was prepared and characterized as previously described.^4^ In brief, the 1959-sss antibody was reduced using a 60-fold molar excess of tris­(2-carboxyethyl) phosphine (TCEP; Thermo Fisher Scientific) in phosphate-buffered saline (PBS; Sigma-Aldrich) at pH 7.4. The reaction was allowed to proceed overnight at room temperature (RT). Following reduction, the antibody was incubated in 100 mM phosphate buffer (pH 7.4) with a 100-fold molar excess of 5,5′-dithiobis­(2-nitrobenzoic acid) (DTNB; Sigma-Aldrich) under the same conditions. The reaction was halted by passing the resulting 1959-sss/DTNB complex through a G25 Sephadex column pre-equilibrated with PBS containing 5% sucrose and 10% *N*,*N*-dimethylacetamide (DMA; Sigma-Aldrich). To conjugate DM4, the DTNB-modified 1959-sss antibody was incubated overnight at RT with a 10-fold molar excess of thiol-maytansinoid DM4 in PBS supplemented with 5% sucrose and 10% DMA. The reaction was then quenched by adding a 500-fold molar excess of iodoacetamide (Sigma-Aldrich). Unconjugated DM4 was removed by size-exclusion chromatography using a G25 Sephadex column equilibrated with PBS/5% sucrose/10% DMA, under isocratic conditions at a flow rate of 1 mL/min. The final ADC concentration was determined by UV/vis spectrophotometry using an extinction coefficient (ε_280_) of 1.6 M^–1^ cm^–1^.

The resulting ADC were characterized by SDS-PAGE and hydrophobic interaction chromatography (HIC) to determine the drug-to-antibody ratio (DAR). Analyses were conducted using an Agilent 1100 liquid chromatography system equipped with a diode array detector (DAD). The stationary phase was a MAbPac HIC-Butyl column (4.6 × 100 mm, 5 μm), maintained at 25 °C (±1 °C). The chromatographic run lasted 20 min and utilized gradient elution to resolve three key components: the free antibody, the antibody conjugated to one molecule of DM4, and the antibody conjugated to two molecules of DM4. Mobile phase A consisted of 2 M ammonium sulfate in 0.1 M phosphate buffer (pH 7.0), while mobile phase B was 0.1 M phosphate buffer (pH 7.0), following the approach described by Tumey.^22^

### 2.4 Stable Lentiviral Transduction

hNB CTRL and hNB LGALS3BP cell lines were obtained by stable lentiviral transduction starting from the hNB parental cell line, as previously described.^14^ Briefly, lentivirus particles were produced by transient cotransfection of the packaging cell line HEK 293T with a three-plasmid expression system using Lipofectamine 2000 (Invitrogen, Thermo-Fisher, Waltham, MA, USA). Lentiviral plasmid pLenti-CMV-PURO-hLGALS3BP for stable overexpression of human LGALS3BP cDNA was obtained as previously described.^14^ Briefly, HEK 293T cells were incubated for 6 h with the transfection reagents. Lentiviral particles were collected after 48 and 72 h and filtered through 0.45 μm pore sterile filters (Corning, NY, USA). The day before the first cycle of transduction, 1.5 × 10^5^ hNB cells were plated. After 24 h, cells were incubated for 6 h with media containing viral particles together with 4 μg/mL polybrene to increase transduction efficiency. After the first cycle of transduction, cells were washed and cultured with fresh medium overnight before a second cycle of transduction the day after. Cells were then allowed to grow under standard growth conditions for 72 h before being put under selection with 1 μg/mL puromycin (Sigma-Aldrich). Selection was maintained throughout all experiments that were performed. LGALS3BP levels were assessed through quantitative real-time PCR (qRT-PCR), Western blotting, and ELISA analysis.

### 2.5 Western Blotting Analysis

Human neuroblastoma cells (5 × 10^5^) were seeded in complete medium, and after 48 h, they were lysed in RIPA buffer (50 mM Tris/HCl pH 7.6, 150 mM NaCl, 1% NP-40, 0.5% Na-deoxycholate, 0.1% SDS, 1 mM EDTA, pH 8) containing protease inhibitor cocktail (Sigma-Aldrich), phosphatase inhibitor cocktail (Roche, Thermo-Fisher), and Na_3_VO_4_ (Sigma-Aldrich). Lysates were clarified by centrifugation at 13,000 rpm for 30 min at 4 °C. Equal amounts of protein lysates (20 μg per sample), previously heated at 95 °C for 5 min, were subjected to SDS-PAGE electrophoresis and then electro-transferred to nitrocellulose membranes for Western blot analysis. Membranes were blocked for 1 h at RT with 5% nonfat dry milk in PBS with 0.1% Tween20. Membranes were then incubated overnight at 4 °C with the following primary antibodies: antihuman LGALS3BP (goat polyclonal; 1:1000, #AF2226; R&D Systems, MN, USA) and anti-β-actin (mouse monoclonal; 1:40,000, #A5441; Sigma-Aldrich). After three washes in PBS-0.1% Tween20, the membranes were hybridized with horseradish peroxidase (HRP)-conjugated secondary antibodies (goat or mouse; Biorad, CA, USA). Detection of signal bands was performed with a Clarity Western ECL substrate (#1705061; Biorad). Images of membranes were acquired with an UvitecFire reader (Cambridge, UK) and analyzed with Alliance Uvitec software (Cambridge, UK).

### 2.6 Quantitative Reverse Transcription Polymerase Chain Reaction (qRT-PCR)

A total of 5 × 10^5^ human neuroblastoma cells were cultured in complete medium for 48 h, and then, total RNA was isolated from collected cells by an RNeasy kit (#74136; Qiagen, Germany). One microgram of total RNA was reverse transcribed to cDNA by using a high-capacity cDNA reverse transcription kit (#4368814; Applied Biosystems, MA, USA) according to the manufacturer’s instructions. Realtime qPCR was performed with a CFX96 Touch Real-Time PCR Detection system (Biorad) using SsoAdvanced Universal SYBR Green supermix (#1725271; Biorad), according to the manufacturer’s instructions. The primers used at a final concentration of 350 nM were hLGALS3BP FOR 5′-GAA CCC AAG GCG TGA ACG AT-3′, hLGALS3BP REV 5′-GTC CCA CAG GTT GTC ACA CA-3′; h-β-actin FOR 5′-CAG CTC ACC ATG GAT GAT GAT ATC-3′, h-β-actin REV 5′-AAG CCG GCCTTG CAC AT-3′. Each sample analysis was performed in triplicate. As a negative control, a no template control was performed. The following PCR program was used: 95 °C for 30 s, 40 cycles of denaturation at 95 °C for 15 s, and annealing/extension at 57 °C for 30 s. To verify the specificity of the amplification, a melting curve analysis was performed immediately after the amplification protocol. qRT-PCR results were calculated using the ΔΔCt method and normalized using human β-actin as the reference gene.

### 2.7 Immunofluorescence and Confocal Imaging

For confocal live cell staining, 2 × 10^5^ hNB CTRL or LGALS3BP were plated on glass coverslips in 12-well plates and cultured under standard growth conditions. After 24 h, cells were incubated for 90 min at 37 °C with 10 μg/mL anti-LGALS3BP antibody 1959 in PBS Ca^2+^/Mg^2+^ with 3% bovine serum albumin (BSA). Cells were then fixed with 4% paraformaldehyde for 10 min at RT and stained with the antihuman AlexaFluor-488 conjugated secondary antibody (1:200, #A11013; Invitrogen) and Hoechst3342 (1:500; Sigma-Aldrich) for 30 min at RT in the dark. Confocal images were acquired using a Zeiss LSM800 inverted confocal microscope system (Carl Zeiss, Germany).

### 2.8 In Vivo Mouse Models and Treatments

NOD.Cg-Prkdcscid Il2rgtm1Wjl/SzJ (NSG) mice were purchased from Jackson Laboratory and bred in the animal facility of CAST, University “G. D’Annunzio” of Chieti-Pescara. Mice were maintained at a temperature of 24–26 °C under pathogen-limiting conditions as required. Cages, bedding, and food were autoclaved before use, and food and water were provided ad libitum. Animal care and experimental procedures were approved by the Ethics Committee for Animal Experimentation of the Institute in accordance with Italian law (Authorization no. 1118/2020-PR). The animal health status was monitored daily, and body weight was measured once a week during experiments. For HPLC method optimization and preliminary analyses, healthy female NSG mice (8 weeks old) were sacrificed, and the lungs, liver, kidneys, and serum were collected.

#### 2.8.1 hNB CTRL and hNB LGALS3BP NSG Pseudometastatic Model

For the therapeutic pseudometastatic experiment, 8 week old female NSG mice were injected intravenously (lateral tail vein) with 1 × 10^4^ hNB CTRL or LGALS3BP cells. After 7 days, mice were randomly divided into groups (*n* = 6) that received vehicle (PBS) or ADC 1959-sss/DM4 (10 mg/kg; twice weekly i.v. administration; three total administrations). After 18 days postinjection (4 days after the last treatment), blood was collected through intracardiac puncture to obtain serum, and lungs were harvested, fixed in 10% neutral buffered formalin, paraffin-embedded, and sectioned at a 5 μm thickness for histological analysis. To optimize the detection of microscopic metastases and ensure systematic, uniform, and random sampling, samples were transversally cut into 2.0 mm thick parallel slabs, with the first cut randomly positioned within the first 2 mm of the organ, resulting in 5–8 slabs per lung and kidney and 6–8 slabs per liver. The slabs were then embedded cut-surface down, and sections were stained with Haematoxylum and Eosin (H&E) for metastasis analysis. Two pathologists independently evaluated slides, and images were acquired using a NanoZoomer scanner (Hamamatsu). To quantify the extent of metastatic lesions at each time point, the percentage of metastatic area relative to the total liver area was assessed using the area annotation tool in NDP.view2 software.

For acute treatments, 14 days postinjection of 1 × 10^4^ hNB CTRL or LGALS3BP cells, mice were randomly divided into groups (*n* = 3) that received single i.v. administration of PBS or 1959-sss/DM4 (10 mg/kg). Tumor-free (TF) mice (*n* = 1) were treated with PBS or 1959-sss/DM4 as controls. After 72 h from treatment, mice were sacrificed, and mouse sera, lungs, and livers were collected, instantly frozen in liquid nitrogen, and stored at −80 °C for further analysis.

#### 2.8.2 SKNAS NSG Pseudometastatic Model

To investigate the pharmacokinetics of DM4 and *S*-Met-DM4 following 1959-sss/DM4 administration, 8 week old female NSG mice were injected via the lateral tail vein with 1 × 10^6^ SKNAS cells. After 3 weeks, mice received a single 10 mg/kg dose of 1959-sss/DM4 and were sacrificed at predefined time points: 0, 8, 16, 24, 36, 48, 56, 72, 96, and 168 h postadministration (*n* = 2 mice for time point). Sera, lungs, livers, and kidneys were collected, with half of each organ fixed, embedded, sectioned, and H&E-stained for metastasis analysis as before, while the remaining portion was snap-frozen for homogenization and analyte extraction. For efficient tissue homogenization, organs were finely minced immediately upon collection as an initial sample processing step before snap freezing in liquid nitrogen.

#### 2.8.3 OVCAR-3 NSG Subcutaneous Xenograft Model

An OVCAR-3 xenograft model was established to investigate the pharmacokinetics of DM4 and *S*-Met-DM4 following mirvetuximab soravtansine administration. OVCAR-3 cells (1 × 10^7^) suspended in 200 μL of PBS and Matrigel (1:3, *v*/*v*) were subcutaneously injected into the right flank of an 8 week old female NSG mouse. When the tumor reached an average volume of approximately 100 mm^3^, the mouse received a single dose of mirvetuximab soravtansine (2.5 mg/kg) and was sacrificed at a predefined time point up to 32 h post-treatment. The tumor, liver, and serum were collected; organs were gently minced, snap-frozen in liquid nitrogen, and stored at −80 °C until further processing.

### 2.9 Immunohistochemistry (IHC)

Sections were immunostained with antihuman LGALS3BP (#AF2226, R&D). Microwave pretreatment (10 min) in citrate buffer (pH 6.0) was performed for antigen retrieval. The biotinylated horse antigoat IgG secondary antibody (BA-9500, Vector Laboratories) and the HRP Detection IHC kit (ab93677, Abcam) were used to detect the antigen. After incubation with the chromogen 3,3-diaminobenzidine DAB (K3468, Dako), slides were counterstained in hematoxylin (CATHE-MM, Biocare Medical) and scanned with a NanoZoomer scanner from Hamamatsu. Images were acquired with NDP.view 2 software.

### 2.10 ELISA

To evaluate the LGALS3BP cell media concentration, 5 × 10^5^ human neuroblastoma cells were cultured in complete medium for 48 h before supernatants were collected. Secreted human LGALS3BP levels were evaluated by sandwich ELISA that was performed as follows: Maxisorp 96-well plates (Nunc, Thermo-Fisher) were coated with murine anti-LGALS3BP antibody SP2 (at 2 μg/mL) overnight at 4 °C.^11^ After blocking with 1% bovine serum albumin (BSA) in PBS for 1 h at RT, 100 μL of cell culture media or recombinant LGALS3BP (15–1000 ng/mL) was added and incubated for 1 h at RT. After three washes with PBS-0.05% Tween20 (*v:v*), humanized anti-LGALS3BP antibody 1959 (at 1 μg/mL) was incubated for 1 h at RT. For the detection, after three washes with PBS-0.05% Tween20, antihuman IgG-HRP (#A01070; Sigma-Aldrich) was added (1:5000) and incubated for 1 h at RT. After three washes, stabilized chromogen was added for at least 10 min in the dark before stopping the reaction with the addition of 1 N H_2_SO_4_. The resulting color was finally read at 450 nm with an ELISA plate reader (Tecan, Switzerland).

To assess the pharmacokinetics of 1959-sss/DM4 in vivo, a custom ELISA-based quantification was performed on serum samples from SKNAS pseudometastatic mice collected at specific time points (8, 16, 24, 32, 48, 56, 72, 100 h) from mice treated with a dose of 10 mg/kg of body weight. Based on the weights recorded at the time of injection, the total ADC administered ranged from approximately 269 to 354 μg per mouse. Serum concentrations of intact ADC were then interpolated and converted to total circulating levels by assuming a blood volume of 1.5 mL per animal.

NUNC Maxisorp 96-well plates (Thermo Fisher Scientific) were coated overnight at 4 °C with 1 μg/mL mouse anti-DM4 monoclonal antibody in PBS. The following day, wells were blocked with 1% (*w:v*) BSA in PBS for 1 h at room temperature (RT). Mice serum samples, diluted 1:1000 in PBS, or 1959-sss/DM4 standards (1–60 ng/mL) were added (100 μL per well) and incubated for 1 h at 1 RT. After three washes with PBS containing 0.05% (*v:v*) Tween-20, detection was performed using an antihuman IgG-HRP conjugate (#A01070, Sigma-Aldrich) diluted 1:5000 in PBS, followed by 1 h incubation at RT. Following another wash step, stabilized chromogen was added and incubated for 10 min in the dark. The reaction was then stopped with 1 N H_2_SO_4_, and the absorbance was measured at 450 nm using a Tecan microplate reader (Männedorf, Switzerland).

### 2.11 Analysis of DM4 and *S*-Me-DM4 in Tissue Extracts

Stock solutions of DM4 and *S*-Met-DM4 were prepared at a concentration of 1 mg/mL by dissolving 1 mg of each compound in 1 mL of DMA. To ensure complete solubilization, these solutions were sonicated in an ultrasonic bath for 2 min at 20 °C.

Blank tissue samples (lung, liver, and kidney) from mice were utilized to establish calibration curves. After homogenizing the tissues in lysis buffer, known amounts of DM4 and *S*-Met-DM4 were spiked into the homogenates at concentrations ranging from 0.04 to 20 μg/mL. For both standard and ex vivo samples, entire organs were weighed and homogenized with lysis buffer at a 1:5 (*w*:*v*) ratio for 1 min at low speed in an ice bath with a tissue raptor and incubated for 30 min to allow complete lysis. Extraction was performed using ethyl acetate:methanol (7.5:1, *v*) at a 1:1 (*v:v*) ratio with the sample, followed by centrifugation at 10,000 rpm for 10 min to recover the organic phase. This step was repeated three times, with all organic phases pooled and subjected to an additional centrifugation at 10,000 rpm for 15 min at 4 °C. The resulting supernatant was dried in a SpeedVac for 2 h, reconstituted in DMA, sonicated for 10 min, and centrifuged at 14,000 rpm for 15 min at 4 °C before chromatographic analysis.

Tissue extracts containing DM4 and *S*-Met-DM4 were analyzed using an HPLC method previously described.^23^ Briefly, an Agilent 1100 liquid chromatography system was used (Agilent Technologies, Waldbronn, Germany), featuring a solvent pump, an online degasser, a thermostated autosampler with a column compartment, and a diode array detector (DAD).

The column used is a C18 reversed-phase column (GraceSmart RP18, 4.6 × 150 mm, 5 μm; Grace, Deerfield, IL, USA) thermostated at 40 °C (±1 °C). Quantitative measurements were performed at 254 nm. The mobile phase consisted of Milli-Q water and methanol in a 25:75 (*v*:*v*) ratio, with both solvents acidified using 0.10% (*v:v*) formic acid. Isocratic elution was conducted at a flow rate of 1 mL/min, with an injection volume of 20 μL. The method was rigorously validated in accordance with international guidelines, with assessments covering linearity, selectivity, precision, trueness (both intraday and interday), ruggedness, and limits of detection (LOD) and quantification (LOQ).

### 2.12 Software Analysis and Statistical Evaluation

Data acquisition and processing was performed using Chemstation Software (Agilent). For statistical analysis and method validation parameters, GraphPad Prism 9.0 Software was used (GraphPad Software, Inc., San Diego, CA, USA). For quantitative and pharmacokinetic data was used Excel and the add-on “PK Solver”. For the evaluation of statistical significance of metastatic areas, the unpaired *t*-test was used and *p* < 0.05 was considered as statistically significant.

## 3 Results

### 3.1 ADC Generation, Characterization, and Payload Analysis

The linker-less noninternalizing ADCs targeting LGALS3BP were obtained through the site-specific conjugation of the ravtansine (DM4) maytansinoid to the engineered mAb 1959-sss, as previously described.^9^

To evaluate the Drug Antibody Ratio (DAR), we optimized a Hydrophobic Liquid Chromatography (HIC) method using mobile phase A composed of 1.5 M ammonium sulfate with 0.1 M sodium phosphate (pH 7.0) and mobile phase B composed of 0.1 M sodium phosphate (pH 7.0). Various gradient profiles were tested to fine-tune the resolution between peaks, and the final gradient conditions are detailed in Table S1. The corresponding chromatogram clearly distinguishes the free antibody and the ADC conjugated with two DM4 molecules, shown in Figure S1A. As visible, retention time of the naked Ab is 12.9 min, instead 14.3 min for the ADC with two molecules of DM4. The presence in 13.5 min of an unquantifiable peak is due to the little presence of DAR 1, data ensured in previous synthesis before optimization.

The drug–antibody ratio (DAR) was determined by calculating the area percentage of each chromatographic peak using the following equation

$$DAR=\frac{\left[\left({AUC}_{0}\%\times 0\right)+\left({AUC}_{1}\%\times 1\right)+\left({AUC}_{2}\%\times 2\right)\right]}{100}$$

where AUC_0_% is related to the free antibody and AUC_1_% and AUC_2_% represent the antibody conjugated to one or two molecules, respectively. As reference control for retention time, free antibody was injected before conjugation. The ADC batch produced and used in this work displayed an average DAR equal to 2, and their purity was confirmed by SDS-PAGE, reported in Figure S1B. Detailed parameters related to the HIC analysis are reported in the Supporting Information (in Table S1 is reported the gradient program used for the separation of the three part of the ADC; meanwhile in Figure S1A, there is the overlapping of the chromatograms related to naked Ab and ADC with two molecules of payload, and in Figure S1B, there is Coomassie stain of an SDS-PAGE ensuring what was find with HIC).

With the aim to analyze ADC payload in tumor samples, we developed a chromatographic method able to detect and quantify both DM4 and its metabolite *S*-Met-DM4. First, the tissue extraction protocol was optimized to obtain the highest chromatographic sensitivity. Analytes were spiked in blank tissues, and different weight-to-volume (*w*:*v*) ratios (1:2, 1:5, and 1:10) were tested for homogenization, using a Tissue Rupture device at low speed for 1 min to minimize drug loss. The 1:5 ratio offered the best balance between analyte recovery and matrix effects, making it the preferred choice. This, visible in Figure S2 (showing the chromatogram obtained through fortification of tissue samples), is due to matrix interference in 1:2 (*w:v* ratio), differently from that in 1:5 and 1:10, which were almost equal. To follow GC principles, a lower lysis buffer volume was chosen. Although sonication was initially considered to enhance cell lysis, it resulted in sample oxidation and was therefore excluded from the final protocol.

Regarding analyte extraction, early attempts with protein precipitation (PP) through acetonitrile (AcN) or dimethylacetamide (DMA) in a 1:3 (*v:v*) ratio yielded poor analyte recovery. As a result, a liquid–liquid extraction (LLE) strategy was adopted. Various homogenization solvents were evaluated, including 10 mM Tris–HCl, PBS (pH 7.5), acidified water (pH 4.0), alkaline water (pH 10), a 50:50 (*v*:*v*) water/methanol mixture, and lysis buffer with and without *N*-ethylmaleimide (NEM). Alkaline water (pH 10.0) caused complete tissue saponification and was excluded. Ultimately, lysis buffer without NEM provided the highest recovery and was selected as the optimal solvent. For LLE, two extraction solvent systems were compared: ethyl acetate:methanol (7.5:1, *v*:*v*) and ethyl acetate:methyl butyl ether (1:1, *v:v*). The latter caused a significant increase in HPLC system pressure, making ethyl acetate:methanol (7.5:1, *v*:*v*) the preferred option. In keeping with Green Chemistry principles, we minimized the extraction solvent volume from a 3.4:1 ratio to a 1:1 ratio with the homogenate without compromising efficiency. Drying time was also optimized, revealing that a 2 h SpeedVac process effectively removed solvent while preventing heat-induced degradation of the analytes. Moreover, instead of using only a representative portion of the tissue, the entire sample was homogenized to maintain reproducibility in drug quantification.

Finally, to determine the optimal experimental conditions for validating our novel HPLC method, extraction efficiency for each tissue type was evaluated by comparing the integrated chromatographic peak areas of samples spiked with DM4 or *S*-Met-DM4 either before (i.e., spiked directly into the lysis buffer) or after the homogenization process, as required by ICH guidelines.^24–26^ In accordance with these guidelines, a recovery test was performed, as reported in Table S2, highlighting the recovery fortifying tissue samples before and after sample extraction/treatment. The results demonstrate that the extraction method is reproducible and minimizes the analyte loss during the optimized procedure. Given the use of different biological matrices, the test was repeated across all matrices employed for method validation, revealing consistent recovery values.

### 3.2 Method Validation

The analytical method was validated in accordance with international guidelines for linearity, selectivity, precision, trueness, and sensitivity. The chromatographic conditions used were the same as those of our previous work.^23^ Both DM4 and its primary metabolite *S*-Met-DM4 were obtained after being spiked in blank homogenized tissues. Analytical figures of merit for method validation obtained on lung tissue, the first analyzed, are shown in Table S3. Calibration curves for both DM4 and its primary metabolite *S*-Met-DM4 were linear over the range of 0.06 to 20 μg/mL, with a correlation coefficient (*R* ^2^) of 0.9928, ensuring that the method reliably quantified the analytes even at low concentrations.

For DM4, the mean retention time was established at 3.87 min. Intraday and interday precision were confirmed by examining quality control (QC) samples at low, medium, and high concentration levels. For instance, the intraday precision (*n* = 33) showed a standard deviation (ST DEV) of 0.03, with retention times consistently near 3.87 min, while interday precision (*n* = 55) demonstrated similar reliability with minor variations (RSD values of 0.74% to 1.71%). The method’s trueness was validated with QC samples showing slight negative biases (around −7.6% to −11.2% for DM4), yet these values were within acceptable limits. Sensitivity metrics further confirmed the robustness of the method, with a limit of detection (LOD) of 0.04 μg/mL and a limit of quantification (LOQ) of 0.06 μg/mL. The calibration slopes (48.30 for intraday and 47.70 for interday) and intercepts (approximately 1.345–1.487) reinforced the method’s linear response.

*S*-Met-DM4, with a mean retention time of 5.37 min, exhibited a comparable performance. The intraday and interday precisions were confirmed by ST DEVs of 0.07 and 0.12, respectively, with relative standard deviations (RSDs) ranging from 1.25% to 2.32%. Slight variations in retention time (minimum values near 5.25 min and maximum values up to 5.80 min) were observed. The slopes for *S*-Met-DM4 were around 14.44 to 14.57, with intercepts close to 6.395 and 6.318 and *R* ^2^ values of 0.995 and 0.992. Together, these parameters underline the method’s high sensitivity, precision, and reliability for both analytes and are reported in Table S4. To ensure robustness and reproducibility, the method was validated across different tissue types: for not only lung but also liver, kidney, and solid tumor. Blank matrices were compared to matrices spiked with DM4 and *S*-Met-DM4 of either liver, kidney, or solid tumor to avoid interferences due to the change of the matrix and to ensure the correct response of the previously optimized protocol. More information about validation parameters for each matrix is reported in Table S4.

Once linearities were achieved, *t*-test was investigated to check out intercept and slope trends matching all tissues. Pairwise comparisons between the four matrices were performed using independent two-sample *t*-tests. Six pairwise tests were conducted to evaluate potential differences between all possible combinations (lung vs kidney, lung vs liver, lung vs tumor, kidney vs liver, kidney vs tumor, liver vs tumor) of matrices for the drug and its main metabolite. Confidence level between various matrices is allowable, being above 99% (for more details, see Table S5). At the end, the method was successfully validated in lung, liver, kidney, and solid tumor tissues. Furthermore, to demonstrate the applicability of the new method to a different DM4-based ADC, we conducted a preliminary evaluation in a subcutaneous xenograft model of human ovarian OVCAR-3 cells treated with the clinically approved mirvetuximab soravtansine.^27,28^ Although limited to a single animal and time point, the method successfully detected both DM4 and its metabolite *S*-Met-DM4 at 32 h postintravenous administration, confirming the potential of this method for analyzing multiple ADCs sharing the same payload.

### 3.3 Specific Antitumor Activity and Accumulation of the Anti-LGALS3BP ADC in Neuroblastoma Pseudometastatic Mouse Models

To assess the specificity and robustness of the method, the previously described ADC 1959-sss/DM4 was employed for in vivo detection and monitoring of its released payloads, DM4 and *S*-Met-DM4. The primary human neuroblastoma cell line hNB, which lacks endogenous LGALS3BP expression, was used to generate an engineered variant ectopically overexpressing a high level of human LGALS3BP. This model enabled a direct comparison of ADC targeting in LGALS3BP-positive versus LGALS3BP-negative tumors. As shown in Figure 1A, ELISA assay of hNB LGALS3BP cell medium exhibited significantly elevated ectopic expression of the protein compared to parental cells (hNB CTRL), whose expression was confirmed by intracellular protein analyses and mRNA levels (Figure 1B,C). Furthermore, the engineered cells displayed both intracellular and secreted LGALS3BP levels comparable to those of SKNAS neuroblastoma cells, which endogenously express the protein. Importantly, we confirmed 1959 antibody specificity trough confocal microscopy analysis in which cell surface and cell milieu were specifically labeled in hNB LGALS3BP but not in control cells (Figure 1D).

**Figure 1:**
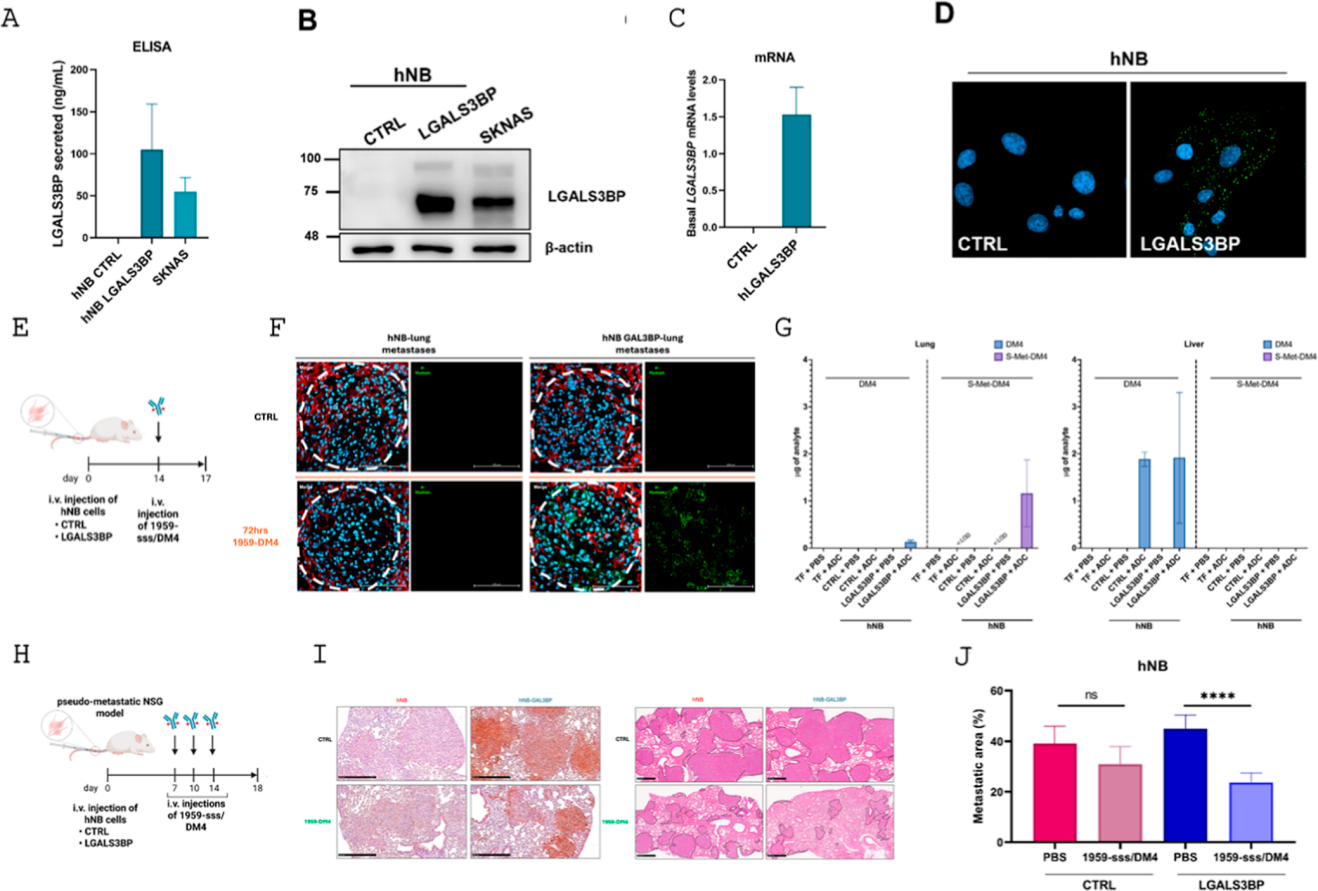
(A) Secreted levels (ng/mL) of LGALS3BP evaluated by ELISA assay in hNB CTRL, hNB LGALS3BP, or SKNAS neuroblastoma cells. (*n* = 2) (B) Western blotting images showing intracellular protein levels of LGALS3BP in hNB CTRL, hNB LGALS3BP, or SKNAS neuroblastoma cells. Equal amounts of protein were loaded for each sample. β-actin was used as loading control. (*n* = 2) (C) Histogram showing transcriptional levels of LGALS3BP in hNB CTRL and hNB LGALS3BP cells normalized to the housekeeping gene (β-actin). (*n* = 3) (D) Confocal imaging of hNB CTRL and LGALS3BP cells showing LGALS3BP (green) and nuclei (Hoechst). Scale bar: 20 μm. (E) Schematic representation of acute treatment (72 h) of the hNB-induced pseudometastatic experiment: hNB CTRL or LGALS3BP cells were tail-vein injected in NSG mice, and at 14 days postinjection, a single i.v. administration of vehicle (PBS) or 1959-sss/DM4 (10 mg/kg) was performed. Mice were sacrificed after 72 h post-treatment to evaluate biodistribution of 1959-sss/DM4 and abundance s-Met-DM4 in lung metastases. Three mice were used per group. Tumor-free (TF) mice were treated with vehicle (PBS) or 1959-sss/DM4 (10 mg/kg) as controls (*n* = 1). (F) Representative sections of lung metastases induced by hNB CTRL or hNB LGALS3BP cells in NSG mice i.v. treated in acute for 72 h with PBS or 1959-sss/DM4 immunostained with antihuman (green) for the detection of 1959-sss/DM4, with anti-CD31-105 (red) as the endothelial marker, and Hoechst (blue) for nuclei. Merge of immunostaining or antihuman staining is reported for each condition. Scale bar: 100 μm. (G) Histogram of the abundance (μg/mL) of DM4 (Left panel) and *S*-Met-DM4 (Right panel) metabolites obtained through HPLC detection 72 h post-treatment in lung metastases and livers. TF stands for tumor-free, CTRL stands for hNB LGALS3BP-negative and LGALS3BP stands for hNB LGALS3BP-positive. (H) Schematic representation of the therapeutic hNB-induced pseudometastatic experiment: hNB CTRL or LGALS3BP cells were tail-vein injected into NSG mice, and at 7 days postinjection, mice were treated with vehicle (PBS) or with 1959-sss/DM4 (10 mg/kg; twice weekly; three total injections). Six mice were used per group. (I) Immunohistochemistry staining of human LGALS3BP (left) or hematoxylin/eosin (right) at the end of the experiment (day 18) on lung metastases induced by hNB CTRL or LGALS3BP cells in NSG mice treated with vehicle (PBS) or with 1959-sss/DM4. Scale bars: 100 μm for LGALS3BP staining IHC; 500 μm for hematoxylin/eosin staining. (J) Percentages of metastatic areas detected in lungs of mice at the end of the experiment (day 18). Statistical significance (*t*-test) is reported: ns, not significant; ****p < 0.0001.

The selective targeting of the 1959-sss/DM4 ADC and payload accumulation were evaluated in the pseudometastatic NSG mice model where hNB-derived lesions (LGALS3BP positive or negative) were analyzed after ADC or PBS administrations. Specifically, after 72 h from treatment, animals were sacrificed, and lung and liver tissues were harvested for antihuman immunostaining on tissue sections and HPLC analysis to quantify DM4 and its metabolite, *S*-Met-DM4 (Figure 1E). We first confirmed the specific accumulation of the anti-LGALS3BP ADC in hNB LGALS3BP-induced lesions, as observed by the antihuman immunostaining performed on lung sections (Figure 1F).

Chromatographic analysis of lung tissues from PBS-treated, nontumor-bearing mice yielded clean baselines with no detectable interference at the analyte retention times, thereby affirming the specificity and reliability of our analytical method. In ADC-treated groups, clear and distinct peaks for both DM4 and *S*-Met-DM4 were observed (Figure 1G and Table 1). Notably, lung tissues, recognized as the primary site for hNB cell attachment, from mice bearing LGALS3BP-positive tumors exhibited significantly elevated levels of both analytes compared to those from LGALS3BP-negative tumors, where DM4 and *S*-Met-DM4 were below the limit of detection, suggesting that the ADC facilitates efficient payload release and accumulation at the intended target site (Figure 1G, left panel). Conversely, in liver tissues, which serve as a central hub for drug metabolism and clearance, both DM4 and *S*-Met-DM4 were consistently detected across all ADC-treated groups (Figure 1G, right panel). Interestingly, at 72 h post-treatment, nearly all DM4 that reached the metastatic lung tissue resulted to be metabolized into *S*-Met-DM4; meanwhile, the liver exhibited relatively higher levels of DM4 than the targeted lung tissues in both LGALS3BP-positive and -negative tumor-bearing mice. In these cases, the presence of DM4 and the absence of *S*-Met-DM4 suggests that DM4 may rapidly reach the target tissue and subsequently be delivered to the liver for metabolic elimination.

**Table 1:** Total Extracted Amount (μg) of DM4 and Its Metabolite *S*-Met-DM4 through HPLC Detection 72 h Post-treatment in Lung and Liver Metastases^a^

Treatment Group	Lung		Liver	
1959-sss/DM4	DM4 (μg)	*S*-Met-DM4 (μg)	DM4 (μg)	*S*-Met-DM4 (μg)
LGALS3BP-positive hNB	0.14 ± 0.03	1.16 ± 0.71	1.91 ± 1.39	
LGALS3BP-negative hNB	<LOD	<LOD	1.88 ± 0.15	
non-tumour-bearing (PBS control)	<LOD	<LOD		

a LOD, Limit of Detection.

Additionally, the therapeutic activity of 1959-sss/DM4 was validated in a treatment setting where NSG mice were treated starting 7 days after hNB cell injection (Figure 1H), a time point at which metastases were already detectable (Figure S3). Treatment was administered twice weekly with a total of three doses at 10 mg/kg, while control animals received vehicle (PBS). A potent, target-dependent ADC effect was observed, as evidenced by a significant reduction in metastatic lesion area, especially in mice injected with LGALS3BP-expressing hNB cells but not in those bearing parental hNB tumors (Figure 1I,J). Overall, these findings confirm the specificity of the HPLC analytical method in detecting DM4 and *S*-Met-DM4 in complex biological matrices, also providing compelling evidence for the ADC’s selective tumor targeting in the metastatic neuroblastoma preclinical model.

### 3.4 Comparative Pharmacokinetic Behavior of Intact 1959-sss/DM4 and Its Released Payload in Tumor Tissues and Systemic Circulation

To better evaluate the potential of the novel HPLC method developed, we employed a second pseudometastatic model of neuroblastoma using the SKNAS cell line, which endogenously expresses a considerable amount of LGALS3BP (Figure 1A,B). In this setting, we evaluated the biodistribution and pharmacokinetics of DM4 and its metabolite *S*-Met-DM4 over a nine-time-point period (8, 16, 24, 36, 48, 56, 72, 100, and 168 h) post single administration of 1959-sss/DM4 ADC. As the primary site of metastasis of SKNAS cells is the liver, ( Table S6, Figure S4), hepatic tissues were collected, and analytes were extracted and analyzed by HPLC. While DM4 was detectable only during early time points (from 8 to 24 h), suggesting a swift uptake followed by rapid metabolic conversion or clearance (Figure 2A, left; Table S7) and reaching peak concentration at 8 h, by contrast, the *S*-Met-DM4 metabolite demonstrated a delayed kinetic profile, reaching peak levels at 32 h and remaining detectable from 16 to 56 h post treatment (Figure 2A, right; Table S7). Lung and kidney tissues, which are secondary metastatic organs, also exhibited ADC-derived analytes, albeit at substantially lower levels ( Table S7), in line with the reduced number of metastatic lesions observed (data not shown).

**Figure 2:**
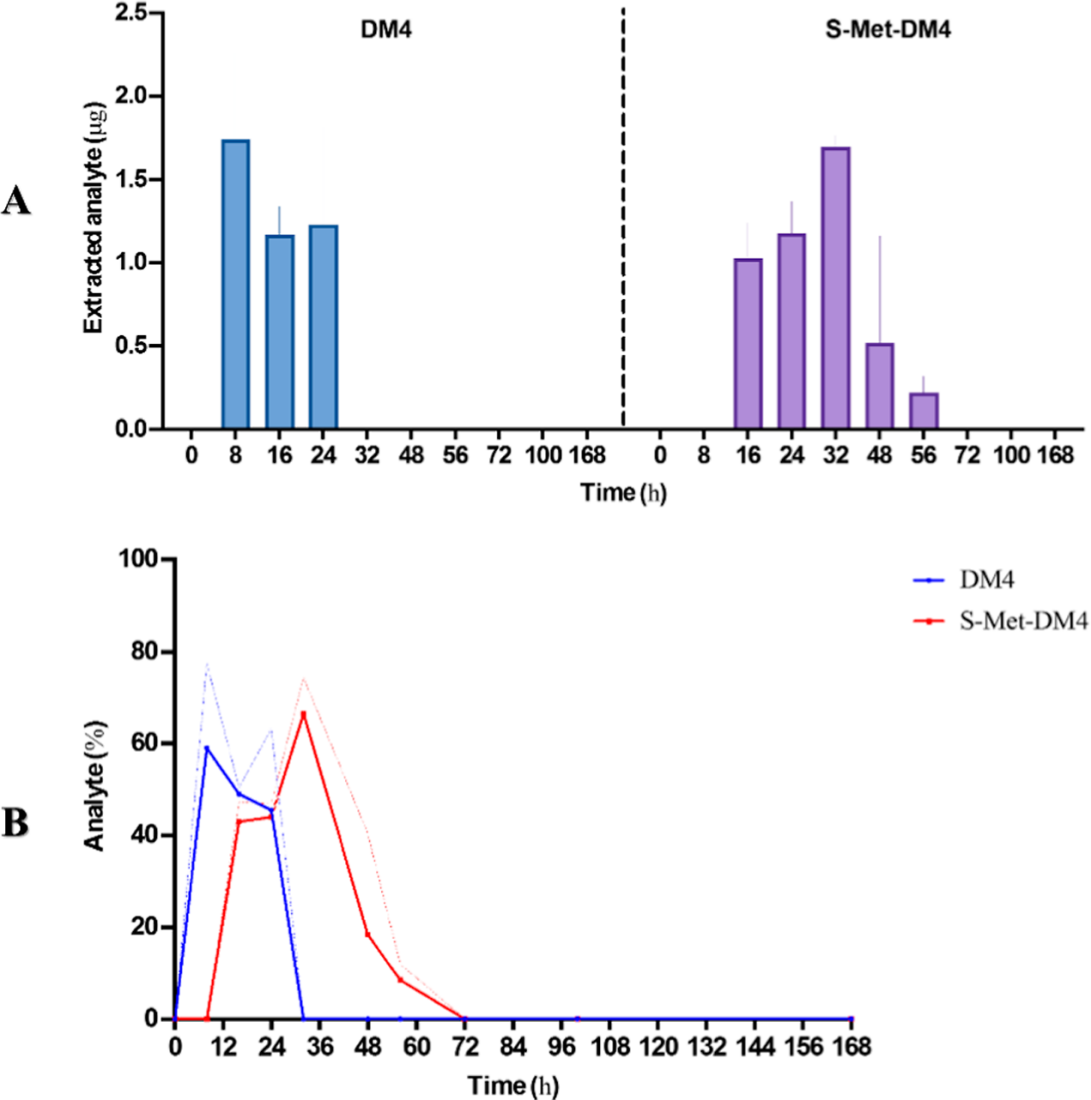
(A) Histogram showing accumulation (μg) of DM4 (left panel) and *S*-Met-DM4 (right panel) through HPLC detection at the indicated time points in mouse metastatic livers. (B) Dispersion graph showing recovered analyte (%) of DM4 (blue) and *S*-Met-DM4 (red) through HPLC detection at the indicated time points in mouse livers.

Serum pharmacokinetics of the intact 1959-sss/DM4 ADC was evaluated by ELISA in tumor-bearing mice. The data revealed a rapid decline in circulating ADC levels after injection (Figure S5A and Table S8). At 8 h, the intact ADC was still detectable in the bloodstream, with circulating quantities corresponding to roughly 25–30% of the total administered dose ( Figure S5B). This suggests that during the initial distribution phase, a substantial fraction of the ADC payload is released at the tumor lesions, consistent with DM4 accumulation observed by HPLC at 8 h. Over the following 24 to 48 h, serum levels of intact ADC continued to decrease, with only a minor fraction of the original dose remaining in circulation, approximately below 10%. These findings support the hypothesis that DM4 is predominantly released from the ADC moiety within the first 24 h postadministration, suggesting a relatively rapid release kinetic.

The pharmacokinetic data provide a compelling picture of the ADC’s behavior ( Figure S5B) and its payload (Figure 2B) release dynamics in vivo. In the metastatic tissues, the released DM4 payload displays a rapid elimination profile, with a half-life of approximately 5.6 h and a *T*_max_ of 8 h, indicating that its release from the ADC occurs quickly upon reaching the tumor microenvironment. In contrast, the metabolite *S*-Met-DM4 shows a distinctly delayed *T*_max_ of 32 h and a prolonged mean residence time of around 28.5 h, suggesting how, following the initial release of DM4, metabolic conversion to *S*-Met-DM4 occurs gradually, resulting in a sustained presence of the active cytotoxic species within the tumor tissue, although the overall exposure (AUC ∼ 63.7 μg·h) for *S*-Met-DM4 is comparable to that of free DM4 (AUC ∼ 56.6 μg·h) (Table 2).

**Table 2:** Pharmacokinetic Parameters of In Vivo, Cancer Metastasis, ADC-Delivered DM4 and *S*-Met-DM4

Parameter	Unit	DM4	*S*-Met-DM4
Lambda_*z*	1/h	0.12	0.07
*t*_1/2_	h	5.60	9.72
*T*_max_	h	8.00	32.00
*C*_max_	μg	1.87	1.70
*C*_0_	μg	2.99	1.03
Clast_obs/*C*_max_		0.02	0.17
AUC_0–*t*_	μg h	56.32	59.68
AUC_0‑inf_obs_	μg h	56.56	63.74
AUC_0–*t*/0‑inf_obs_		1.00	0.94
AUMC_0‑inf_obs_	μg h^2^	772.63	1816.45
MRT_0‑inf_obs_	h	13.66	28.50
Vz_obs	(μg)/(μg)	36.43	56.07
Cl_obs	(μg)/(μg)/h	4.51	4.00
Vss_obs	(μg)/(μg)	61.59	113.99

### 3.5 Greenness of the Method

The analytical method’s environmental sustainability was assessed using tools such as AGREE, AGREEprep, BAGI, and MoGAPI, which confirmed its alignment with green chemistry principles. A notable limitation identified was the offline collection of animal samples, necessitated by ethical guidelines for obtaining pharmacokinetic parameters.^29–33^ The method’s use of ethyl acetate and minimal methanol enhances its environmental profile as these solvents are considered less toxic alternatives in chromatographic applications. The isocratic elution and 15 min run time contribute to energy efficiency and facilitate scalability to more sensitive configurations, such as liquid chromatography coupled with mass spectrometry. The method’s simplicity allows for the quantification of the drug and its main metabolite across various tissues without interference from different biological matrices, using standard instrumentation that requires less energy and does not necessitate highly specialized personnel. The straightforward sample preparation and analysis procedures suggest potential applicability in clinical settings. The almost overlap of most of the used tools means a good applicability, following various aspects of GC, as shown in Figure 3.

**Figure 3:**
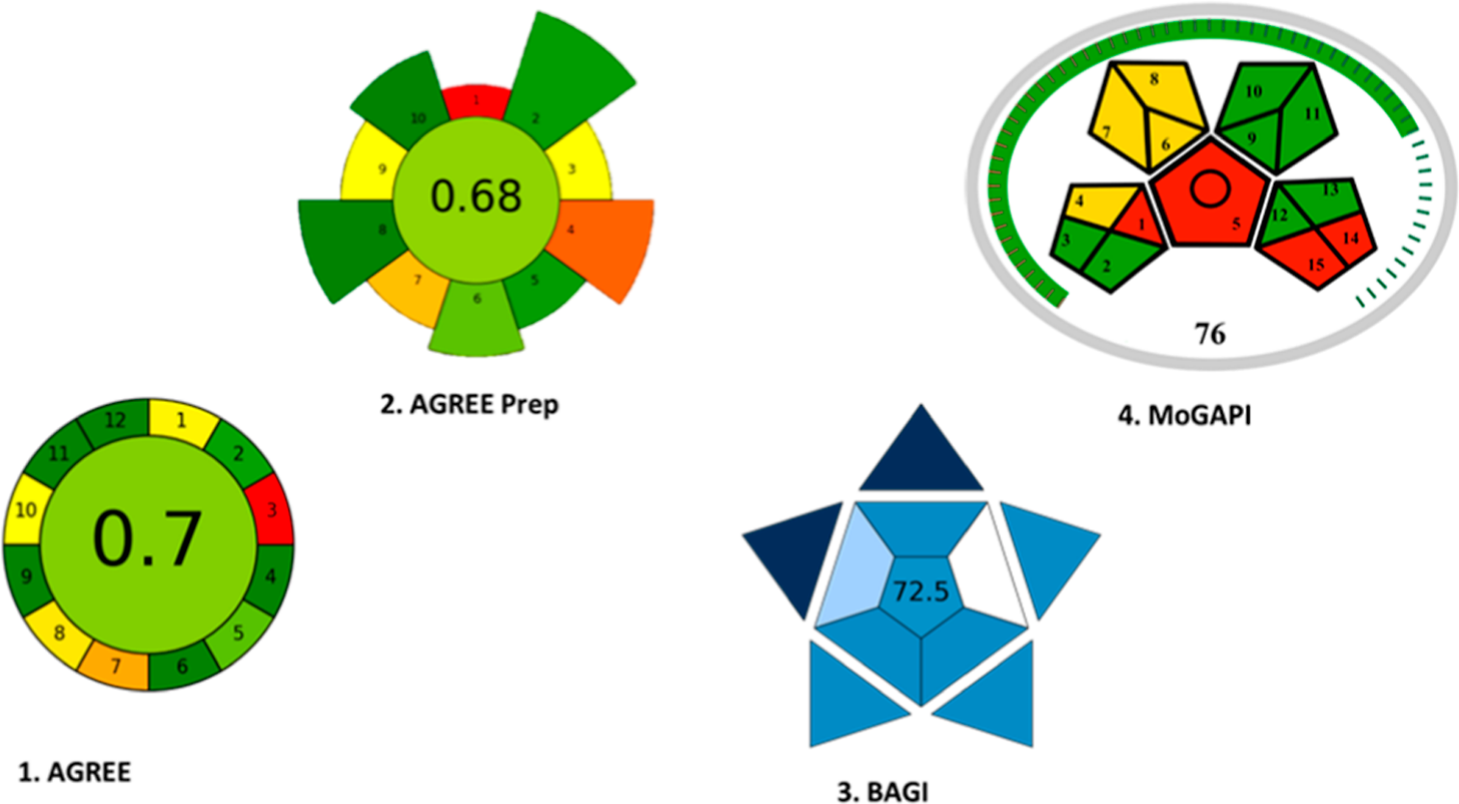
Pictograms related to the herein reported procedure.

## 4 Discussion

In this study, we present a validated HPLC–DAD method that enables the simultaneous quantification of the DM4 payload and its active metabolite, *S*-methyl-DM4, in biological matrices. To our knowledge, this is the first validated method capable of simultaneously quantifying both DM4 and its primary metabolite, *S*-methyl-DM4, in multiple biological matrices by using HPLC–DAD. A comprehensive review of the literature revealed that existing quantification methods for these compounds rely almost exclusively on LC–MS/MS platforms, particularly in preclinical and pharmacokinetic studies. No peer-reviewed reports describing the use of HPLC–DAD for DM4 or *S*-methyl-DM4 detection in tissues such as liver, lung, or kidney were found.^34–36^ Furthermore, many LC–MS/MS methods show variable extraction recoveries and typically require complex and costly instrumentation. In contrast, our method achieves high and consistent recovery across all tested matrices using a more accessible detection system while maintaining strong linearity, precision, and reproducibility. These features position this HPLC–DAD approach as a novel, technically accessible, and reproducible alternative capable of resolving both analytes directly in solid tissues, without the need for derivatization, radiolabeling, or protein tagging.

Following analytical validation, the HPLC–DAD assay was employed to profile the payload accumulation on tumor sites of the noninternalizing 1959-sss/DM4 ADC, which is currently under preclinical development.^9,12,14–16^ To demonstrate the validity and specificity of this method, we utilized an LGALS3BP-negative neuroblastoma cell line (hNB), engineered to ectopically express LGALS3BP, to establish a robust system for specifically detecting the target-dependent ADC-delivered DM4 and *S*-Met-DM4 accumulation within tumor lesions. HPLC analysis revealed highly selective payload accumulation in LGALS3BP-positive lesions. In hNB LGALS3BP-bearing mice, DM4 and *S*-Met-DM4 peaks were detected in lung tissues 72 h post-treatment, whereas both analytes remained below the limit of detection in parental hNB controls and vehicle-treated animals. Conversely, liver samples, reflecting systemic clearance, consistently contained both analytes in ADC-treated groups regardless of the LGALS3BP status. In addition, HPLC data were consistent with the in vivo therapeutic effect of 1959-sss/DM4. Our data show a significant reduction in lung metastatic burden in hNB LGALS3BP-injected mice treated with 1959-sss/DM4, confirming both the functional specificity and therapeutic efficacy of this ADC.

Subsequently, we decided to better profile the behavior of the released payload over time. In the pseudometastatic SKNAS model, where metastatic lesions localize preferentially to the liver, we observed a selective accumulation of DM4 in tumor-bearing mice, peaking between 8 and 24 h after ADC administration. Importantly, *S*-methyl-DM4 levels rose more gradually and persisted over time, consistent with intracellular metabolism of the released payload and diffusion throughout the tumor lesions due to the “bystander” effect. The relative pharmacokinetic evaluation revealed a clear sequential release and turnover of the maytansinoid payload. The parent compound is released rapidly once the ADC localizes in tumor tissue. Over time, metabolic conversion gives rise to the *S*-methylated metabolite, which accumulates more slowly but remains detectable for an extended period, sustaining antitumor activity beyond the initial DM4 release. At the same time, the kinetics of the intact ADC in serum collected from tumor-bearing mice shows a rapid decline, aligning with the observed payload accumulation in tumor tissue. This indicates that payload release rapidly occurs within the tumor microenvironment, strongly suggesting that payload delivery is targeted rather than systemically persistent.

To ensure the possibility of using this validated method on different ADCs with the same payload, we applied it to the clinically approved DM4-based ADC mirvetuximab soravtansine,^27,28^ which shares the same chemistry and payload as 1959-sss/DM4 ( Figure S6). Although this evaluation was limited to a single mouse and one time point, the method successfully detected both analytes, thereby confirming its robustness and establishing a strong rationale for its use in future studies across multiple DM4-based ADCs.

## 5 Conclusions

To our knowledge, this is the first report of a fully HPLC–DAD-based approach applied to the analysis of a maytansinoid-conjugated ADC, with potential applications in preclinical pharmacokinetics and biodistribution studies. These findings not only confirm the specificity of 1959-sss/DM4 but also provide a framework for future comparisons with other maytansinoid-based ADCs. Assessing payload accumulation in vivo across different conjugation strategies may also clarify the impact of linker–linkerless chemistry on delivery efficiency. Furthermore, it lays the groundwork for head-to-head comparisons of different conjugation strategies and linker chemistries and, given its isocratic format, can be readily adapted to LC–MS/MS detection for enhanced sensitivity and multiplexed metabolite profiling.

## Supplementary Material



## Data Availability

All relevant data are provided within the manuscript.

## Notes

Gradient profile used for HIC analysis; HIC chromatogram; S1B: SDS-PAGE; chromatogram obtained from analysis of spiked lung; recovery percentage of DM and *S*-Me-DM4 for each tissue fortified before and after extraction; analytical parameters for intra- and inter- day in lung tissue; analytical linearity parameters for each matrix used (lung, kidney, liver, tumor); two-tailed *t*-test for DM4 and *S*-Me-DM4; hNB CTRL and hNB LGALS3BP-induced metastases in lungs mice after 7 days from cell injection (hematoxylin/eosin staining and anti-LGALS3BP immunostaining); percentage of liver metastatic area after treatment with the ADC at different time points; hematoxylin and eosin (H&E) stained sections illustrating SKNAS-induced metastatic lesions in livers; recovered amount (μg) of DM4 and *S*-Me-DM4 from mice tissues (lung, liver, and kidney) treated with a single dose of 1959-sss/DM4 and sacrificed after different time points post-treatment; amount (μg) of intact 1959-sss/DM4 from serum of mice treated with a single dose of the ADC and sacrificed after different time points post-treatment using the ELISA method; histogram showing percentage of amount of the intact ADC using ELISA; dispersion graph with recovery of the intact ADC over time; and chromatogram showing real sample analysis of the extracted tumor subjected to intravenous administration of the clinically approved DM4-based ADC (PDF)

## References

[1] Dumontet C., Reichert J. M., Senter P. D., Lambert J. M., Beck A. (2023). Antibody–drug
conjugates come of age in oncology. Nat. Rev.
Drug Discovery.

[2] Kumari S., Raj S., Babu M. A., Bhatti G. K., Bhatti J. (2024). S Antibody–drug
conjugates in cancer therapy: Innovations, challenges, and future
directions. Arch. Pharmacal Res..

[3] Lambert J. M., Berkenblit A. (2018). Antibody–drug conjugates for
cancer treatment. Annu. Rev. Med..

[4] Ponziani S., Di Vittorio G., Pitari G., Cimini A. M., Ardini M., Gentile R., Iacobelli S., Sala G., Capone E., Flavell D. J., Ippoliti R., Giansanti F. (2020). Antibody–Drug
Conjugates: The New Frontier of Chemotherapy. Int. J. Mol. Sci..

[5] Kamath A. V., Iyer S. (2016). Challenges and advances
in the assessment of the disposition of antibody-drug
conjugates. Biopharm. Drug Dispos..

[6] Sevilla-Carrillo L., García-Tercero E., Alonso-Moreno C., Moya-Lopez C. (2025). Antibody Drug Conjugates (ADCs): Shaping the Future
of Precision Oncology. Adv. Anticancer Agents
Med. Chem..

[7] Villacampa G., Morgado P. C., Carità L., Navarro V., Pascual T., Dienstmann R. (2024). Safety and
efficacy of antibody-drug conjugates plus
immunotherapy in solid tumours: A systematic review and meta-analysis. Cancer Treat. Rev..

[8] Capone E., Iacobelli S., Sala G. (2021). Role of galectin 3 binding protein
in cancer progression: a potential novel therapeutic target. J. Transl. Med..

[9] Giansanti F., Capone E., Ponziani S., Piccolo E., Gentile R., Lamolinara A., Di Campli A., Sallese M., Iacobelli V., Cimini A., De Laurenzi V., Lattanzio R., Piantelli M., Ippoliti R., Sala G., Iacobelli S. (2019). Secreted galectin-3
binding protein as a therapeutic target for non-internalizing antibody–drug
conjugates. Cancer Res..

[10] Piccolo E., Tinari N., Semeraro D., Traini S., Fichera I., Cumashi A., La Sorda R., Spinella F., Bagnato A., Lattanzio R., D’Egidio M., Di Risio A., Stampolidis P., Piantelli M., Natoli C., Ullrich A. (2013). LGALS3BP,
lectin galactoside-binding soluble 3 binding protein, induces vascular
endothelial growth factor in human breast cancer cells and promotes
angiogenesis. J. Mol. Med..

[11] Traini S., Piccolo E., Tinari N., Rossi C., La Sorda R., Spinella F., Bagnato A., Lattanzio R., D’Egidio M., Di Risio A., Tomao F., Grassadonia A., Piantelli M., Natoli C., Iacobelli S. (2014). Inhibition
of Tumor Growth and Angiogenesis by SP-2, an Anti-Lectin, Galactoside-Binding
Soluble 3 Binding Protein (LGALS3BP) Antibody. Mol. Cancer Ther..

[12] Capone E., Lamolinara A., Pastorino F., Gentile R., Ponziani S., Di Vittorio G., D’Agostino D., Bibbò S., Rossi C., Piccolo E., Iacobelli V., Lattanzio R., Panella V., Sallese M., De Laurenzi V., Giansanti F., Sala A., Iezzi M., Ponzoni M., Ippoliti R., Iacobelli S., Sala G. (2020). Targeting Vesicular
LGALS3BP by an Antibody-Drug Conjugate as Novel Therapeutic Strategy
for Neuroblastoma. Cancers.

[13] Capone E., Perrotti V., Cela I., Lattanzio R., Togni L., Rubini C., Caponio V. C. A., Lo
Muzio L., Colasante M., Giansanti F., Ippoliti R., Iacobelli S., Wick M. J., Spardy
Burr N., Sala G. (2024). Anti-LGALS3BP antibody-drug conjugate treatment induces
durable and potent antitumor response in a preclinical model of adenoid
cystic carcinoma. Oral Oncol..

[14] Cela I., Capone E., Pece A., Lovato G., Simeone P., Colasante M., Lamolinara A., Piro A., Iezzi M., Lanuti P., De Laurenzi V., Ippoliti R., Iacobelli S. (2025). LGALS3BP antibody-drug conjugate enhances tumor-infiltrating lymphocytes
and synergizes with immunotherapy to restrain neuroblastoma growth. J. Transl. Med..

[15] Dufrusine B., Capone E., Ponziani S., Lattanzio R., Lanuti P., Giansanti F., De Laurenzi V., Iacobelli S., Ippoliti R., Mangiola A., Trevisi G., Sala G. (2023). Extracellular LGALS3BP: a potential
disease marker and actionable
target for antibody-drug conjugate therapy in glioblastoma. Mol. Oncol..

[16] Keinänen O., Sarrett S. M., Delaney S., Rodriguez C., Dayts E. J., Capone E., Sauniere F., Ippoliti R., Sala G., Iacobelli S., Zeglis B. M. (2023). Visualizing galectin-3
binding protein expression with immunoPET. Mol.
Pharmaceutics.

[17] Erickson H. K., Park P. U., Widdison W. C., Kovtun Y. V., Garrett L. M., Hoffman K., Lutz R. J., Goldmacher V. S., Blättler W. A. (2006). Antibody-maytansinoid conjugates
are activated in targeted
cancer cells by lysosomal degradation and linker-dependent intracellular
processing. Cancer Res..

[18] Nguyen T. D., Bordeau B. M., Balthasar J. P. (2023). Use of
Payload Binding Selectivity
Enhancers to Improve Therapeutic Index of Maytansinoid-Antibody-Drug
Conjugates. Mol. Cancer Ther..

[19] Staudacher A. H., Brown M. P. (2017). Antibody drug conjugates
and bystander killing: is
antigen-dependent internalisation required?. Br. J. Cancer.

[20] Ricci E. M., Dainese E., De Laurenzi V., Lovato G., Sala G., Locatelli M., Perrucci M. (2025). New analytical challenges in characterization
of antibody–drug conjugates. J. Chromatogr.
Open..

[21] Zhu, X. Novel LC-MS Strategies for Enhancing Understanding of Drug Effects and Exploring Disease Mechanisms. Doctoral dissertation, State University of New York at Buffalo, 2024.

[22] Tumey, L. N. Antibody-Drug Conjugates; Springer US, 2020.

[23] Lovato G., Ciriolo L., Perrucci M., Federici L., Ippoliti R., Iacobelli S., Capone E., Locatelli M., Sala G. (2023). HPLC–DAD validated method for the simultaneous quantification
of DM4 and S-methyl-DM4 in biological matrices. J. Pharm. Biomed. Anal..

[24] Center for Drug Evaluation and Research; Center for Veterinary Medicine Bioanalytical Method Validation-Guidance for Industry, Food and Drug Administration, May, 2018.

[25] Validation of Analytical Procedures: Text and Methodology Q2­(R1). International Conference on Harmonization of Technical Requirements for registration of Pharmaceuticals for Human Use, ICH Harmonised Tripartite Guideline 2005, Geneva, 2005.

[26] ICH guideline M10 on bioanalytical method validation and study sample analysis. International Conference on Harmonization of Technical Requirements for registration of Pharmaceuticals for Human Use, ICH Harmonized Tripartite Guideline, 2022.

[27] Matulonis U. A., Vergote I., Moore K. N., Martin L. P., Castro C. M., Gilbert L., Xia Y., Method M., Stec J., Birrer M. J., O’Malley D. M. (2025). Safety
and efficacy of mirvetuximab
soravtansine, a folate receptor alpha (FRα)-targeting antibody-drug
conjugate (ADC), in combination with pembrolizumab in patients with
platinum-resistant ovarian cancer. Gynecol.
Oncol..

[28] Dilawari A., Shah M., Ison G., Gittleman H., Fiero M. H., Shah A., Hamed S. S., Qiu J., Yu J., Manheng W., Ricks T. K., Pragani R. P., Arudchandran A., Patel P., Zaman S., Roy A., Kalavar S., Ghosh S., Pierce W. F., Rahman N. A., Tang S., Mixter B. D., Kluetz P. G., Pazdur R., Amiri-Kordestani L. (2023). FDA Approval
Summary: Mirvetuximab Soravtansine-Gynx for FRα-Positive, Platinum-Resistant
Ovarian. Clin Cancer Res..

[29] Locatelli M., Kabir A., Perrucci M., Ulusoy S., Ulusoy H. I., Ali I. (2023). Green profile tools: current status
and future perspectives. Adv. Sample Prep..

[30] Pena-Pereira F., Wojnowski W., Tobiszewski M. (2020). AGREE—Analytical
GREEnness
metric approach and software. Anal. Chem..

[31] Wojnowski W., Tobiszewski M., Pena-Pereira F., Psillakis E. (2022). AGREEprep-analytical
greenness metric for sample preparation. TrAC,
Trends Anal. Chem..

[32] Mansour F. R., Płotka-Wasylka J., Locatelli M. (2024). Modified GAPI (MoGAPI) tool and software
for the assessment of method greenness: case studies and applications. Analytica..

[33] Manousi N., Wojnowski W., Płotka-Wasylka J., Samanidou V. (2023). Blue applicability
grade index (BAGI) and software: a new tool for the evaluation of
method practicality. Green Chem..

[34] Fu Y., Gorityala S., Li W., Humphries D., Ledvina A., Cape S., Picard F. (2022). Sensitive LC-MS/MS
quantification of unconjugated maytansinoid DM4 and its metabolite
S-methyl-DM4 in human plasma. Bioanalysis.

[35] Liu Y., Zhou F., Sang H., Ye H., Chen Q., Yao L., Ni P., Wang G., Zhang J. (2017). LC–MS/MS method
for the simultaneous determination of Lys-MCC-DM1, MCC-DM1 and DM1
as potential intracellular catabolites of the antibody-drug conjugate
trastuzumab emtansine (T-DM1). J. Pharm. Biomed.
Anal..

[36] Wei C., Su D., Wang J., Jian W., Zhang D. (2018). LC–MS challenges
in characterizing and quantifying monoclonal antibodies (mAb) and
antibody-drug conjugates (ADC) in biological samples. Curr. Pharmacol. Rep..

