# Supplementary material for: Evaluation of Antibody–Drug Conjugate Performances Using a Novel HPLC–DAD Method for Tumor-Specific Detection of DM4 and S‑Methyl-DM4
Source: ACS Omega. 2025 Oct 15;10(42):50382–93. doi: 10.1021/acsomega.5c07693 (PMC12572975; doi:10.1021/acsomega.5c07693)
Supplement: Supplementary file 1 [file ao5c07693_si_001.pdf]

Evaluation of Antibody-Drug Conjugate Performances Using a Novel HPLC-DAD Method for Tumor-Specific Detection of DM4 and S-Methyl-DM4

Giulio Lovato [a, b, §]; Miryam Perrucci [a, c, §]; Ilaria Cela [a, b]; Alessia Lamolinara [d], Arianna Mercatelli[a, b] Vincenzo De Laurenzi[a, b], Emily Capone [b, e]; Marcello Locatelli [e*] and Gianluca Sala [a, b*]

[a] *Department of Innovative Technologies in Medicine and Dentistry, Center for Advanced Studies and Technology (CAST), University of Chieti-Pescara "G. d'Annunzio", Via dei Vestini 31, Chieti, 66100, Italy.*
[b] *Center for Advanced Studies and Technology (CAST), University of Chieti-Pescara "G. d'Annunzio", Via dei Vestini 31, Chieti, 66100, Italy.*
[c] *Department of Biosciences and Agro-Food and Environmental Technologies, University of Teramo 64100 Teramo, Italy*
[d] *Department of Medicine and Aging Sciences, G. d'Annunzio University of Chieti-Pescara, 66100 Chieti, Italy*
[e] *Department of Science, University of Chieti-Pescara "G. d'Annunzio" 66100 Chieti, Italy*

§ These Authors contributed equally

* Corresponding authors:

g.sala@unich.it

marcello.locatelli@unich.it

Supplementary materials.

Table S1. Gradient used for separation of Ab, Ab conjugated to one molecule of DM4 and AB with two molecules of drug.

HIC Method parameters		
**Time (min)**	**%A**	**%B**
**0**	99	1
**1**	99	1
**13**	15	85
**13.01**	99	1
**20**	99	1

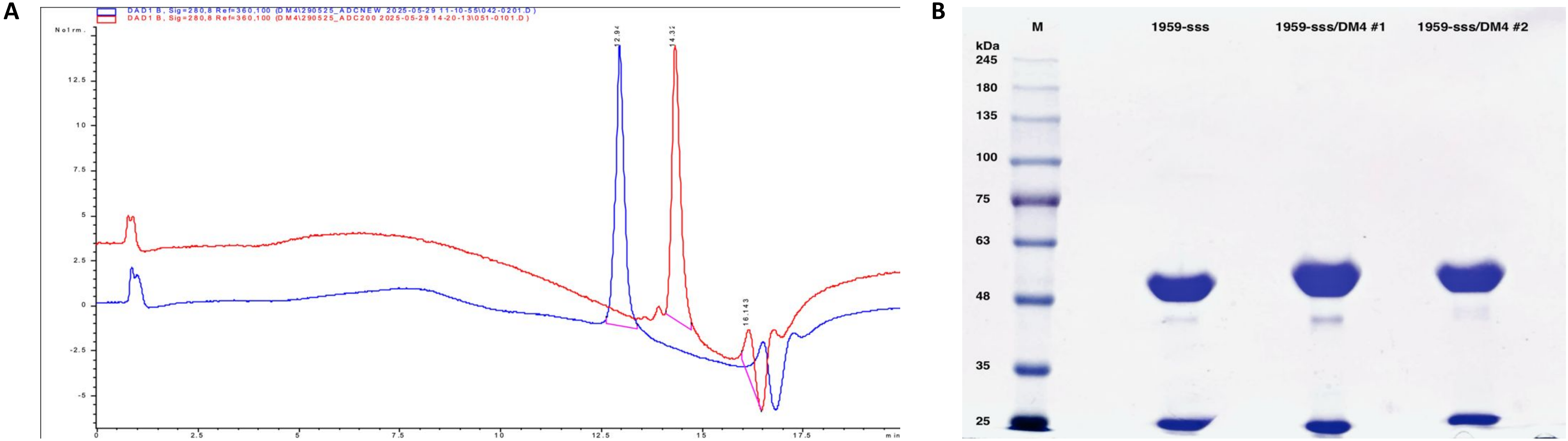

Figure S1. (**A**) Hydrophobic Interaction Chromatography (HIC): overlapping of chromatogram obtained through the analysis of naked 1959-sss (blu line) and chromatogram obtained analyzing 1959-sss/DM4 ADC after its conjugation synthesis (red line). (**B**) Coomassie stain of an SDS-PAGE showing differences in size and purity between naked 1959-sss and two batches of ADC 1959-sss/DM4.

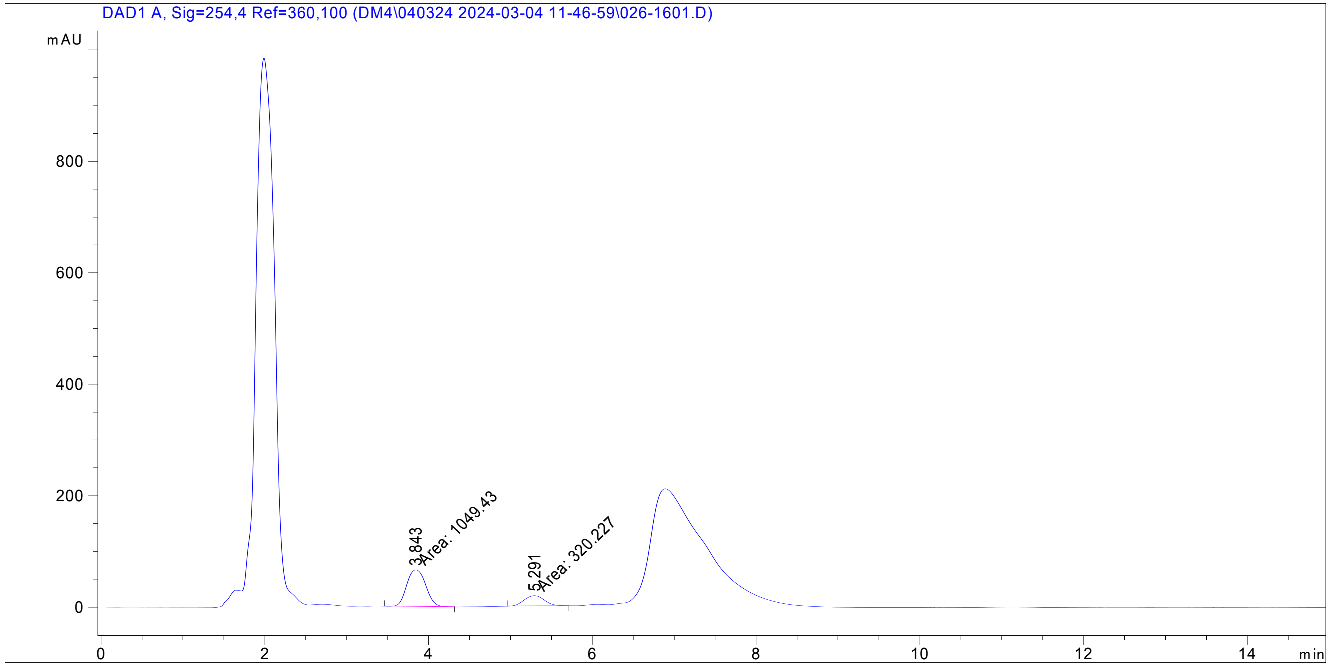

Figure S2. Chromatogram obtained by spiked matrix with 20 µg/ mL of both drugs (DM4 and S-Met-DM4).

Table S2. Recovery percentage of DM4 and S-Met-DM4 for each tissue analyzed (Lung, Kidney, Liver and solid tumour) based on the ratio between the integrated area of chromatographic peak between spiked addition of analytes pre and post homogenization.

	**DM4**			**S-Met-DM4**		
	**Integrated Peak Area**		**% Recovery**	**Integrated Peak Area**		**%Recovery**
**Tissue**	**Pre**	**Post**		**Pre**	**Post**	
**Lung**	551,00	858,00	64,22	161,70	232,40	69,58
**Kidney**	344,05	616,30	55,83	162,90	251,55	64,76
**Liver**	343,00	473,65	72,42	163,85	189,55	86,44
**Tumour**	349,85	579,10	60,41	195,50	243,95	80,14

Table S3. Representative table regarding selectivity, precision, trueness, and sensitivity for method validation in lung tissue.

DM4						S-Met-DM4				
**INTRADAY**			**INTERDAY**			**INTRADAY**			**INTERDAY**	
**Mean Rt (min)**	3.87		**Mean Rt (min)**	3.87		**Mean Rt (min)**	5.37		**Mean Rt (min)**	5.37
**ST DEV**	0.03		**ST DEV**	0.07		**ST DEV**	0.07		**ST DEV**	0.12
**RSD %**	0.74		**RSD %**	1.71		**RSD %**	1.25		**RSD %**	2.32
**Min**	3.82		**Min**	3.76		**Min**	5.25		**Min**	5.13
**Max**	3.93		**Max**	4.09		**Max**	5.53		**Max**	5.80
**n**	33		**n**	55		**N**	33		**n**	55
**LOD**	0.04		**LOD**	0.04		**LOD**	0.04		**LOD**	0.04
**LOQ**	0.06		**LOQ**	0.06		**LOQ**	0.06		**LOQ**	0.06
**Slope**	48.30		**Slope**	47.70		**Slope**	14.44		**Slope**	14.57
**Intercept**	1.345		**Intercept**	1.487		**Intercept**	6.395		**Intercept**	6.318
**R^2**	0.998		**R^2**	0.997		**R^2**	0.995		**R^2**	0.992
**PRECISION**			**PRECISION**			**PRECISION**			**PRECISION**	
**QC Low**	4.18		**QC low**	4.28		**QC Low**	9.79		**QC low**	6.57
**QC Medium**	1.47		**QC Medium**	4.37		**QC Medium**	5.97		**QC Medium**	5.50
**QC High**	2.44		**QC High**	1.90		**QC High**	4.72		**QC High**	7.06
**TRUENESS**			**TRUENESS**			**TRUENESS**			**TRUENESS**	
**QC Low**	-7.64		**QC Low**	-7.97		**QC Low**	-6.34		**QC Low**	-2.81
**QC Medium**	-11.16		**QC Medium**	-10.19		**QC Medium**	-4.65		**QC Medium**	-6.55
**QC High**	-6.03		**QC High**	-4.87		**QC High**	3.21		**QC High**	-1.81

Table S4. Analytical parameters linearity for each matrix.

**DM4**					
		**Lung**	**Kidney**	**Liver**	**Tumor**
	**Polynomial first order**				
**Best fit value**	**Intercept**	-6.230	-6.199	28.03	3.832
	**slope**	49.28	37.67	37.34	32.68
**Std. error**	**Intercept**	2.931	4.284	9.090	3.640
	**slope**	1.503	2.198	4.663	1.867
**95% confidence interval**	**Intercept**	-18.84 to 6.382	-24.63 to 12.24	-11.09 to 67.14	-11.83 to 19.50
	**slope**	42.81 to 55.74	28.21 to 47.12	17.28 to 57.41	24.64 to 40.71
**Goodness of fit**	**Degrees of Freedom**	2	2	2	2
	**R2 (unweighted)**	0.9969	0.9860	0.9379	0.9847
**S-Met-DM4**					
		**Lung**	**Kidney**	**Liver**	**Tumor**
	**Polynomial first order**				
**Best fit value**	**Intercept**	4.887	10.65	25.35	14.77
	**slope**	15.55	15.63	15.91	17.57
**Std. error**	**Intercept**	0.4659	4.402	2.809	3.163
	**slope**	0.2390	2.258	1.441	1.623
**95% confidence interval**	**Intercept**	2.883 to 6.892	-8.294 to 29.59	13.25 to 37.44	1.157 to 28.38
	**slope**	14.52 to 16.58	5.919 to 25.35	9.708 to 22.11	10.59 to 24.55
**Goodness of fit**	**Degrees of Freedom**	2	2	2	2
	**R2 (unweighted)**	0.9995	0.8741	0.9891	0.9287

Table S5. T test two- tailed for drug and its metabolite.

DM4			
**Tissues**	**Slope**	**Intercept**	**T tab**
Lung vs kidney	11.941	0.016	4.303 confidence livel 95%
Lung vs liver	6.674	9.824	9.925 confidence livel 99%
Lung vs tumor	18.967	5.896	22.3327 confidence livel 99.8%
Kidney vs liver	0.175	9.328	
Kidney vs tumor	4.739	4.887	
Liver vs tumor	2.541	6.768	
**S-Met-DM4**			
**Tissues**	**Slope**	**Intercept**	**T tab**
Lung vs kidney	0.096	3.565	4.303 confidence livel 95%
Lung vs liver	0.675	19.681	9.925 confidence livel 99%
Lung vs tumor	3.372	8.466	22.327 confidence livel 99.8%
Kidney vs liver	0.286	7.709	
Kidney vs tumor	1.911	2.082	
Liver vs tumor	2.095	6.849	

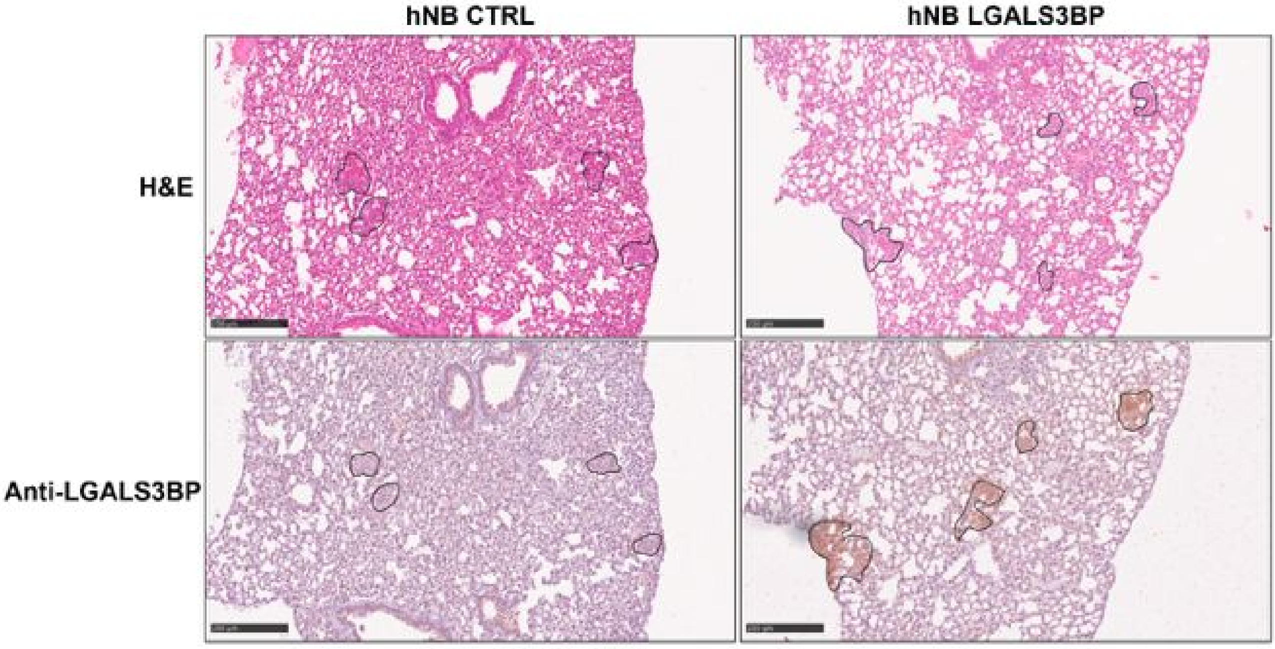

Figure S3. hNB CTRL and hNB LGALS3BP-induced metastases in lungs of NSG mice after 7 days from cell injection. Hematoxylin/eosin staining (upper images) and anti-LGALS3BP immunostaining (lower images) on lungs sections. Scale bar: 250 µm.

Table S6. SKNAS Intravenous (I.V.) Experiment – Time Course Post-Treatment with 1959-sss/DM4: table reports the percentage of liver metastatic area in NSG female mice intravenously inoculated with 1 million SKNAS cells, treated with 1959-DM4, and sacrificed at various time points following treatment.

**From treatment**	**% of metastatic area**
8h #1	52,13
8h #2	71,57
16h #1	62,33
16h #2	60,14
24h #1	61,57
24h #2	64,25
32h #1	74,48
32h #2	74,52
48h #1	41,70
48h #2	81,04
56h #1	74,80
56h #2	64,20
72h #1	50,26
72h #2	71,65
96h #1	51,76
96h #2	59,62

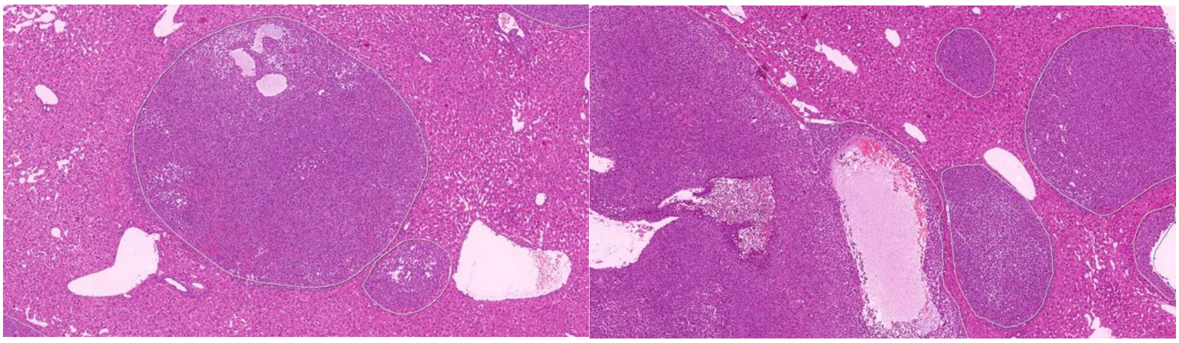

Figure S4. Two representative haematoxylin and eosin (H&E) stained sections illustrating SKNAS-induced metastatic lesions in the livers of these mouse models.

Table S7. Summary of *in vivo* DM4 and S-Met-DM4 HPLC-recovered amount (µg) from mice tissues (Lung, Liver and Kidney) treated with a single dose of 1959-sss/DM4 and sacrificed after different time points post treatment.

**DM4**					**S-Met-DM4**			
	**Extracted Payload (µg)**					**Extracted Payload (µg)**		
**ID**	**Liver**	**Lung**	**Kidney**		**ID**	**Liver**	**Lung**	**Kidney**
**CTRL**	0.00	0.00	0.00		**CTRL**	0.00	0.00	0.00
**8h**	1.74 ± 0.52	0.13 ± 0.18	0.00		**8h**	0.00	0.00	0.00
**16h**	1.17 ± 0.17	0.00	0.00		**16h**	1.03 ± 0.21	0.01 ± 0.01	0.00
**24h**	1.23 ± 0.58	0.00	0.00		**24h**	1.18 ± 0.19	0.00	0.00
**32h**	0.00	0.00	0.00		**32h**	1.70 ± 0.06	0.00	0.00
**48h**	0.00	0.03 ± 0.04	0.00		**48h**	0.52 ± 0.64	0.00	0.16 ± 0.23
**56h**	0.00	0.00	0.00		**56h**	0.22 ± 0.1	0.07 ± 0.1	0.00
**72h**	0.00	0.00	0.00		**72h**	0.00	0.00	0.00
**100h**	0.00	0.00	0.00		**100h**	0.00	0.00	0.00
**168h**	0.00	0.00	0.00		**168h**	0.00	0.00	0.00

Table S8. Summary of ELISA-interpolated amount (µg) of intact 1959-sss/DM4 from serum of mice treated with a single dose of ADC and sacrificed after different time points post treatment.

ID	Intact serum 1959-sss/DM4 (µg)
CTRL	0.00
8h	78.33 ± 1.48
16h	76.60 ± 0.64
24h	63.47 ± 22.15
32h	25.93 ± 4.69
48h	24.01 ± 7.41
56h	7.35 ± 1.79
72h	6.75 ± 8.82
100h	0.62 ± 0.5

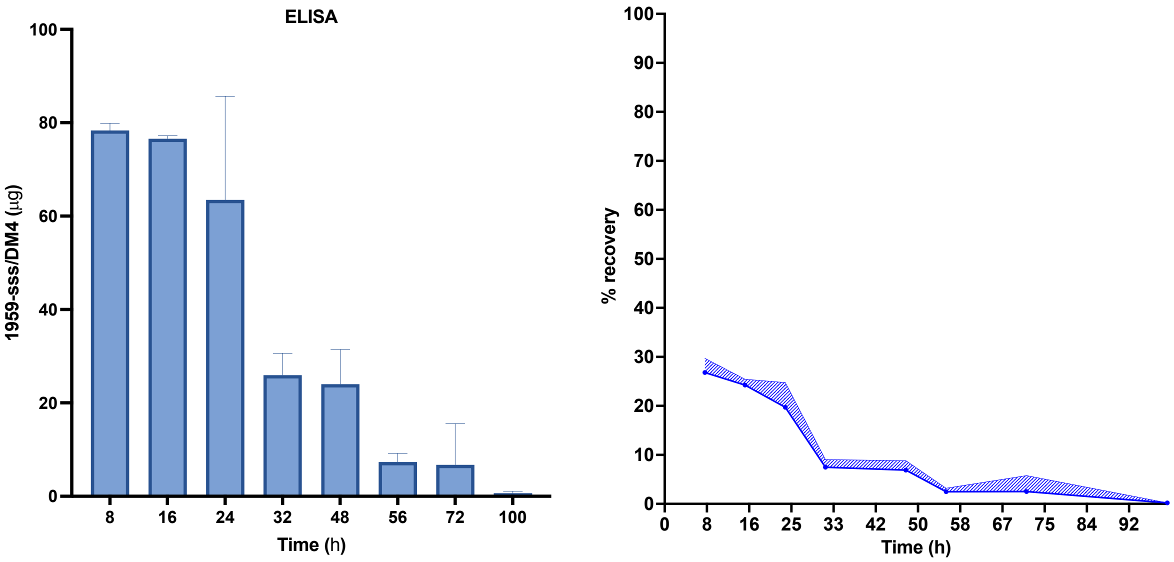

Figure S5. (A) Histogram showing interpolated ELISA-interpolated amount (µg) of intact ADC through ELISA detection at the indicated time-points in mouse livers, **(B)** Dispersion graph showing the recovery (%) of intact ADC during time.

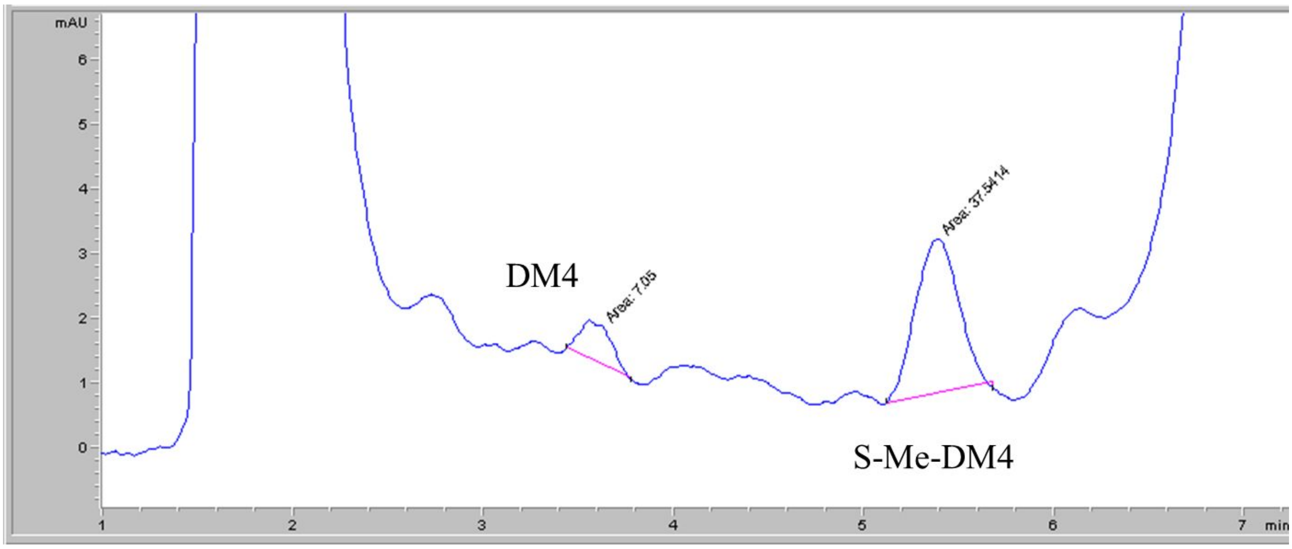

Figure S6. Zoom of the chromatogram obtained after extraction of the tumor tissue growth through OVCAR- 3 and treated with 2.5 mg/ kg of Mirvetuximab soravtansine. As reported, the first peak is due to DM4 and the second bigger peak is its main metabolite, S-Me-DM4. Method was tested on a clinically approved ADC to ensure its work.
